# Change in Motor and Nonmotor Symptoms Severity in a “Real-Life” Cohort of Subjects with Parkinson's Disease

**DOI:** 10.1155/2015/368989

**Published:** 2015-08-20

**Authors:** Adib Jorge de Saráchaga, Amin Cervantes-Arriaga, Rodrigo Llorens-Arenas, Humberto Calderón-Fajardo, Mayela Rodríguez-Violante

**Affiliations:** ^1^Clinical Neurodegenerative Research Unit, National Institute of Neurology and Neurosurgery, 3877 Insurgentes Sur, Tlalpan, 14269 Mexico City, DF, Mexico; ^2^Movement Disorder Clinic, National Institute of Neurology and Neurosurgery, 3877 Insurgentes Sur, Tlalpan, 14269 Mexico City, DF, Mexico

## Abstract

*Background*. Parkinson's disease (PD) is a chronic and progressive disorder. Rates of change in motor symptoms have been more studied compared to nonmotor symptoms. The objective was to describe these changes in a real-life cohort of subjects with PD.* Methods*. A cohort study was carried out from 2011 to 2013. Consecutive patients with PD were recruited from a movement disorders clinic. MDS-UPDRS, PDQ-8, and NMSS were applied to all subjects at an initial evaluation and a subsequent visit (21 ± 3 months). Disease severity was categorized using a recent classification of MDS-UPDRS severity.* Results*. The MDS-UPDRS Part III showed a significant decrease of 7.2 ± 2.31 points (*p* = 0.001) between evaluations. A mean increase of 0.9 ± 0.6 points (*p* = 0.015) in the MDS-UPDRS Part IV was observed. An increase of 14.3 ± 11.4 points (*p* = 0.043) in the NMSS total score was found; when assessed individually, the difference was statistically significant only for the perceptual problems/hallucinations item. Quality of life remained unchanged.* Conclusion*. Motor improvement was observed accompanied by an increase in motor complications possibly as a result of treatment optimization. Nonmotor symptoms worsened as a whole. The overall effect in the quality of life was negligible.

## 1. Introduction

Parkinson's disease (PD) is a chronic and progressive disorder with an estimated prevalence of 2% in adults over age 60 [1]. The study of disease progression and its determinants is of great importance to improve our understanding of the disease in order to optimize treatment [2].

A wide variety of correlations between different subtypes of the disease and the progression of motor and nonmotor symptoms have been reported. A cohort study with an eight-year follow-up found that axial symptoms (gait and postural instability) progress more rapidly than other motor features of PD (tremor, bradykinesia, and rigidity) [3]. Likewise, a study with a nine-year follow-up reported a greater progression of motor scores in subjects with the following characteristics: male gender, older age at diagnosis, akinetic-rigid subtype, and lower baseline motor score [4]. A slower progression of tremor in comparison to other cardinal features of PD has also been reported [5].

On the other hand, nonmotor progression has been less studied. A study derived from the ADAGIO study population demonstrated an increase of 10% in the Movement Disorder Society Unified Parkinson's Disease Rating Scale (MDS-UPDRS) Part I score, which evaluates nonmotor experiences of daily living (nM-EDL), in the placebo group through a nine-month follow-up [6]. Another study reported a rate of progression for nM-EDL of 0.42 points per year, while progression on the motor experiences of daily living (M-EDL) was reported to be of 0.8 points per year [7]. Other reported risk factors for faster progression include orthostatic hypotension and hallucinations [8].

The objective of the present study is to describe the change in motor and nonmotor symptoms assessed by the MDS-UPDRS and the nonmotor symptoms scale (NMSS) in a real-life cohort of subjects with PD after a 21-month follow-up.

## 2. Methods

### 2.1. Participants

A cohort study was carried out from 2011 to 2013. Subjects with PD were recruited from the Movement Disorders Clinic of the National Institute of Neurology and Neurosurgery (Mexico City). Diagnosis was made according to the UK Parkinson's Disease Brain Bank's Criteria by a movement disorder specialist [10].

The study was submitted and obtained approval from the Institutional Review Board and Local Ethics Committee. Full signed consent from all participants was obtained in order to participate in the study.

### 2.2. Assessments

General demographic data and PD history information were collected using a standardized questionnaire. Levodopa equivalent daily dose (LEDD) was also calculated [11]. The Spanish version of MDS-UPDRS, the Parkinson Disease Quality of Life Questionnaire (PDQ-8), and the NMSS were applied to all subjects at two different cut-points (initial evaluation and a follow-up visit at 21 ± 3 months).

The full 65-item MDS-UPDRS was applied by a neurologist with expertise in movement disorders. The MDS-UPDRS consists of four parts: Part I, Non-Motor Experiences of Daily Living; Part II, Motor Experiences of Daily Living; Part III, Motor Examination; and Part IV, Motor Complications [12]. All patients were assessed during their “on” clinical state.

The PDQ-8 is a health status scale covering eight different dimensions of health-related quality of life. Each item is scored using a Likert scale (never, occasionally, sometimes, and always). The PDQ-8 is expressed as a summarized index [13].

The NMSS is a nine-domain scale for the evaluation of nonmotor symptoms in PD (cardiovascular, sleep/fatigue, mood/cognition, perceptual problems/hallucinations, attention/memory, gastrointestinal tract, urinary function, sexual function, and miscellaneous) [14]. Each domain is assessed in terms of severity (from 0: none to 3: severe) and frequency (from 1: rarely to 4: very frequent) in the last month. The score for each domain is obtained by multiplying frequency by severity; total score is the sum of the nine domains.

Disease severity was categorized using the recently published triangulation-based cut-offs classification of MDS-UPDRS severity (Part I: Mild 0–10, Moderate 11–21, and Severe ≥22; Part II: Mild 0–12, Moderate 13–29, and Severe ≥30; Part III: Mild 0–32, Moderate 33–58, and Severe ≥59; Part IV: Mild 0–4, Moderate 5–12, and Severe ≥13) [9].

### 2.3. Statistical Analysis

Descriptive statistics were applied for demographic data. Quantitative data such as MDS-UPDRS, NMSS, and PDQ-8 scores were compared using a *t*-test for related samples. Ordinal data (use of antiparkinsonian drugs) were compared using a McNemar test; when more than two outcomes were present (disease severity) the McNemar-Bowker test was used. A value of *p* < 0.05 for statistical significance was set for all analyses. Data was analyzed using SPSS version 17.

## 3. Results

A total of sixty patients were recruited. Fifty-three patients concluded the follow-up (33 women and 20 men). In all cases, loss to follow-up was due to migration out of the city resulting in unavailability to attend the follow-up visit. The mean age at the initial visit for the final sample was 64.1 ± 14.3 years and the mean disease duration was 9.1 ± 5.4 years. Comparison of treatment schemes between the initial and follow-up visits is shown in Table 1. Only two patients underwent bilateral deep brain stimulation during the study. The frequencies for each severity group according to the MDS-UPDRS at the initial and follow-up visits are shown in Table 2. The comparison of total scores in the MDS-UPDRS, PDQ-8, and NMSS between visits is shown in Table 3.

### 3.1. Change in Motor Symptoms

No statistically significant difference was found in regard to disease severity according to the MDS-UPDRS Part III between the initial and follow-up visits. Most of the patients remained in the mild and moderate disease groups, although there was a slight increase in the mild group as a consequence of clinical improvement in subjects initially classified as moderate severity.

The MDS-UPDRS Part III demonstrated a statistically significant decrease of 7.2 ± 2.3 points (95% CI, 3.1 to 11.2, *p* = 0.001) between the initial and follow-up visits. When compared by disease severity, subjects with mild disease had a mean improvement of 5.7 ± 0.1 points (95% CI, 3 to 8.4, *p* < 0.0001); subjects with moderate disease also showed improvement although statistical significance was not reached (8 ± 3.5 points, 95% CI 4.4 to 20.4, *p* = 0.159).

The m-EDL assessed by MDS-UPDRS Part II did not show any statistically significant change between visits.

In regard to motor complications assessed by the MDS-UPDRS Part IV, a mean increase of 0.9 ± 0.6 points (95% CI, 0.8 to 1.6, *p* = 0.015) was observed. When analyzed by severity, subjects with a mild disease worsened by 0.4 ± 0.2 points (95% CI, 0.1 to 0.8, *p* = 0.017).

No statistically significant association was found between the mean change in the total MDS-UPDRS score and the predominant motor phenotype (*p* = 0.397). When analyzed independently, no association was found between the different MDS-UPDRS parts and predominant phenotype (Part I *p* = 0.787, Part II *p* = 0.286, Part III *p* = 0.578, and Part IV *p* = 0.994). No association was found between motor scores and gender (*p* = 0.427) or disease duration (*p* = 0.941).

### 3.2. Change in Nonmotor Symptoms

No statistically significant change in nonmotor severity as assessed by the MDS-UPDRS Part I was found between visits with most of the patients remaining in the mild severity group. Moreover, the nM-EDL score did not show a statistically significant change between the initial and follow-up visits even when accounting for severity classification. Conversely, all nonmotor symptom domains in the NMSS scale showed an increase in the mean score, as shown in Table 4. An increase of 14.3 ± 11.4 points (95% IC, 0.47 to 27.4, *p* = 0.043) in NMSS total score was found between visits. Even though there was an increase in the score of all domains, the difference was statistically significant only for the perceptual problems and hallucinations item (0.2 ± 0.7 to 0.8 ± 2.1, *p* = 0.044). When analyzing by disease severity according to the MDS-UPDRS Part I score, no statistically significant increase in NMSS total score within groups was found. The MDS-UPDRS Part I and NMSS total scores showed a high correlation (*r* = 0.611, *p* = 0.01).

No statistically significant associations were found between NMSS scores and disease duration (*p* = 0.677), gender (*p* = 0.964), or motor phenotype (*p* = 0.427).

### 3.3. Quality of Life

No statistically significant changes were found in quality of life as assessed by the PDQ-8 between visits.

## 4. Discussion

PD is a progressive neurodegenerative disease. Rates of change in motor and nonmotor symptoms appear to progress differently in a nonlinear fashion with a greater increase in the M-EDL in comparison to the nM-EDL [7]. Progression of the disease usually translates in the severity of the symptomatology. Traditionally, PD severity is assessed using the Hoehn and Yahr staging. In this regard, the MDS-UPDRS severity scale was preferred instead due to the fact that the Hoehn and Yahr scale relies mainly on the motor state. In order to evaluate the impact of nonmotor symptoms a severity classification accounting for them was needed. The recently published cut-off points for PD severity levels based on the MDS-UPDRS had the advantage of including nonmotor symptoms [9].

In the present study, motor symptoms improved after the 21-month follow-up.

The overall improvement of seven points in the MDS-UPDRS Part III can be explained by several factors. Firstly, the study was carried out at a referral center and the initial evaluation was actually the first time the patient was seen at the clinic. As a consequence, the reduction in motor scores might be explained by an optimized pharmacological treatment. It should be pointed out that although the LEDD increased by 120 mg/d, the actual levodopa daily dose was slightly increased. The latter means that no major levodopa dosage adjustments were performed but also that antiparkinsonian drugs were added as expected. For instance, the use of monoamine oxidase inhibitors and the dopamine agonist LEDD was doubled. It also should be emphasized that motor evaluations were performed during the “on” clinical state, in contrast to an “off” state that could be a better index of the disease natural history.

On the other hand, an increase in motor complications such as “on” time with troublesome dyskinesia and motor fluctuations assessed in the MDS-UPDRS Part IV was found. Motor complications related to dopaminergic treatment are expected to increase with disease progression despite better motor scores.

Interestingly, no difference in m-EDL (MDS-UPDRS Part II) was found between visits despite the improvement in motor scales. Moreover, health-related quality of life assessed by the PDQ-8 also failed to show any improvement.

In regard to the nM-EDL, a lack of improvement or worsening is consistent with other reports. Poewe et al. reported a significant worsening of nM-EDL scores in the MDS-UPDRS in the placebo group, but no change in treated patients [6].

On the other hand, a statistically significant increase of 14% in the NMSS total score was observed. Even though every NMSS domain had an increase in its score, only the perceptual changes and hallucinations item had a statistically significant difference. That is, all the nonmotor symptoms worsened, but only the cumulative effect and hallucinations reached statistical significance. The reason why nonmotor symptomatology worsened during the study is not clear. A possible explanation may be that the worsening in individual nonmotor symptoms was not clinically significant and as a consequence proper management was not initiated. For instance, use of antidepressants remained the same despite the increase in the mood domain score. Additionally, some symptoms like hallucinations can be an adverse effect of dopaminergic replacement therapy, as well as a consequence of disease progression.

Our findings oppose the study of Lang et al., which reported a greater decline in m-EDL in comparison to nM-EDL based on MDS-UPDRS Parts I and II [7]. This study had a longer follow-up period (up to 5 years) and only included subjects with PD in early stages. It is possible that discrepancy is the result of a shorter follow-up and the inclusion of subjects with varying degrees of severity. More important is the fact that patients in our study received the best medical treatment in comparison to untreated patients enrolled in a randomized clinical trial setting. As such, our study provides a pragmatic view of the effectiveness of interventions in real-life practice.

It should be highlighted that MDS-UPDRS Part I and NMSS total scores had a high correlation coefficient, but the MDS-UPDRS Part I failed to show any statistically significant difference. Differences in the construct between both instruments may explain this finding. Martinez-Martin et al. reported a strong convergent validity between MDS-UPDRS Part I and NMSS but also a lack of concordance in patients with a high burden of nonmotor symptoms [15].

Our study has several limitations. Although it is expected that MDS-UPDRS scores correlate with the disease duration, the patients assessed in our study had different PD durations. While this issue affects direct extrapolation, it also gives a more objective overview of daily clinical practice. Secondly, patients had different therapeutic schemes at the initial evaluation; thus final outcomes could be influenced by the optimization of the treatment rather than from disease progression. Finally, as mentioned before, all scales were applied during patients' “on” clinical state in order. This might not reflect the natural history of the disease and therapeutic effect should be considered. On the other hand, nonmotor symptoms did not change with treatment as much as motor scores. Also, nonmotor fluctuations were not assessed.

In conclusion, we found a motor improvement during the 21-month follow-up accompanied by an increase in motor complications. Nonmotor symptoms assessed by the NMSS worsened when taken as a whole. Quality of life and M-EDL remain unchanged. Studies assessing motor and nonmotor changes over time in different stages of severity are needed.

## Figures and Tables

**Table 1:** Comparison of drug treatment between initial and follow-up visits.

	Initial visit	Follow-up	*p* value
Use of levodopa^*∗*^	40 (75.5%)	45 (84.9%)	0.063
Levodopa dose (mg)^†^	447.8 ± 399.6	487.5 ± 348.8	0.419
Use of dopamine agonists^*∗*^	31 (41.5%)	28 (52.8%)	0.181
Use of MAOi^*∗*^	4 (7.5%)	8 (15.7%)	0.133
DA-LEDD (mg)^†^	88.2 ± 130.3	160.3 ± 186	0.009
Total LEDD^†^	556.8 ± 410.4	676.3 ± 380.5	0.015
Use of antidepressants^*∗*^	12 (22.6%)	13 (24.5%)	>0.99
Surgery (bilateral DBS)^*∗*^	0%	2 (3.8%)	—

^*∗*^Total (percentage). ^†^Mean ± standard deviation. LEDD: levodopa equivalent daily dose. DA-LEDD: dopamine agonists-levodopa equivalent daily dose. MAOi: monoamine oxidase inhibitor. DBS: deep brain stimulation.

**Table 2:** Parkinson's disease change in severity based on MDS-UPDRS severity scale.

MDS-UPDRS	Initial evaluation	Follow-up	*p* value
MDS-UPDRS Part I			
Mild	33 (62.3%)	35 (66%)	0.751
Moderate	17 (32.1%)	15 (28.3%)	0.789
Severe	3 (5.7%)	3 (5.7%)	0.617
MDS-UPDRS Part II			
Mild	34 (64.2%)	37 (69.8%)	0.579
Moderate	17 (32.1%)	13 (24.5%)	0.422
Severe	2 (3.8%)	3 (5.7%)	<0.99
MDS-UPDRS Part III			
Mild	36 (67.9%)	38 (71.7%)	0.789
Moderate	14 (26.4%)	14 (26.4%)	0.802
Severe	3 (5.7%)	1 (1.9%)	0.617
MDS-UPDRS Part IV			
Mild	49 (92.5%)	45 (84.9%)	0.288
Moderate	4 (7.5%)	8 (15.1%)	0.288
Severe	0% (0)	0% (0)	—

MDS-UPDRS: Movement Disorder Society Unified Parkinson's Disease Rating Scale.

**Table 3:** Comparison of mean scores for MDS-UPDRS, NMSS, and PDQ-8 between initial and follow-up visits according to disease severity^*∗*^.

	Initial evaluation	Follow-up	*p* value
MDS-UPDRS Part I	9.57 ± 6.17	9.11 ± 7.06	0.570
Mild	5.17 ± 2.33	4.96 ± 3.54	0.765
Moderate	15.11 ± 2.93	15.11 ± 2.80	≥0.99
Severe	22.00	33.00	—
MDS-UPDRS Part II	11.32 ± 8.12	11.32 ± 10.21	*≥*0.99
Mild	6.13 ± 3.88	5.58 ± 3.45	0.479
Moderate	18.50 ± 2.44	21.25 ± 5.17	0.185
Severe	38.00	44.00	—
MDS-UPDRS Part III	30.57 ± 16.01	23.40 ± 13.70	*<*0.001
Mild	21.23 ± 7.12	15.53 ± 7.23	<0.0001
Moderate	46.83 ± 7.93	38.83 ± 4.44	0.159
Severe	—	—	—
MDS-UPDRS Part IV	1.11 ± 2.30	2.02 ± 2.93	0.015
Mild	0.48 ± 1.16	0.93 ± 1.36	0.017
Moderate	9.50 ± 0.70	7.00 ± 0.00	0.126
Severe	—	—	—
MDS-UPDRS Total	51.45 ± 22.51	45.84 ± 27.84	0.068
NMSS	38.20 ± 38.96	52.48 ± 50.38	0.043
PDQ-8	21.88 ± 21.20	22.88 ± 20.11	0.575

^*∗*^Mean ± standard deviation. MDS-UPDRS: Movement Disorder Society Unified Parkinson's Disease Rating Scale. NMSS: Nonmotor Symptom Assessment Scale for Parkinson's Disease. PDQ-8: Parkinson's Disease Quality of Life Questionnaire.

**Table 4:** Change in nonmotor symptoms scale score between initial and follow-up visits^**∗**^.

Domain^†^	Initial evaluation	Follow-up	*p* value
Cardiovascular	0.46 ± 1.16	1.02 ± 2.14	0.142
Mild	0.30 ± 1.19	0.34 ± 0.74	0.852
Moderate	0.75 ± 1.16	1.25 ± 2.76	0.598
Severe	0.00	9.00	—
Sleep/fatigue	6.33 ± 8.65	8.19 ± 8.44	0.141
Mild	2.50 ± 4.65	4.23 ± 4.99	0.095
Moderate	11.75 ± 8.54	12.50 ± 7.65	0.795
Severe	28.00	25.00	—
Mood/cognition	9.46 ± 14.40	12.40 ± 17.80	0.172
Mild	2.65 ± 6.80	5.53 ± 10.35	0.104
Moderate	14.00 ± 6.98	15.62 ± 18.11	0.787
Severe	18.00	62.00	—
Perceptual problems/hallucinations	0.17 ± 0.67	0.77 ± 2.10	0.044
Mild	0.07 ± 0.39	0.23 ± 0.86	0.425
Moderate	0.62 ± 1.40	1.12 ± 1.88	0.487
Severe	0.00	11.00	—
Attention	3.26 ± 6.00	5.09 ± 7.64	0.156
Mild	2.26 ± 4.03	3.11 ± 4.70	0.438
Moderate	8.12 ± 11.46	7.25 ± 10.89	0.782
Severe	2.00	36.00	—
Gastrointestinal tract	4.76 ± 6.37	5.56 ± 7.76	0.232
Mild	3.24 ± 4.90	3.00 ± 6.05	0.803
Moderate	4.87 ± 5.33	8.50 ± 8.41	0.280
Severe	12.00	30.00	—
Urinary function	6.17 ± 9.06	8.65 ± 10.94	0.240
Mild	2.72 ± 5.19	4.20 ± 6.53	0.389
Moderate	10.75 ± 11.75	11.75 ± 12.38	0.884
Severe	12.00	36.00	—
Sexual function	2.11 ± 4.69	3.83 ± 7.81	0.298
Mild	0.96 ± 2.38	2.04 ± 5.76	0.370
Moderate	4.87 ± 8.64	6.75 ± 9.37	0.654
Severe	0.00	24.00	—
Miscellaneous	4.80 ± 6.56	6.63 ± 6.38	0.112
Mild	3.24 ± 5.23	5.84 ± 6.13	0.117
Moderate	5.12 ± 4.82	5.37 ± 6.09	0.932
Severe	2.00	21.00	—
Total score	38.2 ± 38.96	52.48 ± 50.38	0.043
Mild	19.32 ± 23.77	28.68 ± 23.06	0.089
Moderate	60.87 ± 33.22	70.12 ± 43.41	0.606
Severe	74.00	254.00	—

^*∗*^Mean ± standard deviation. ^†^Each nonmotor symptom scale domain was subclassified according to the Movement Disorder Society Unified Parkinson's Disease Rating Scale Part I (Non-Motor Experiences of Daily Living) and cut-off values established by Martínez-Martín et al. [9].
